# Supplementary figures and images for: Participant engagement and feedback in microbiome projects: a case of AWI-Gen 2
Source: bioRxiv. 2026 Apr 22:2026.04.20.718838. Preprint. [Version 1] doi: 10.64898/2026.04.20.718838 (PMC13131796; doi:10.64898/2026.04.20.718838)

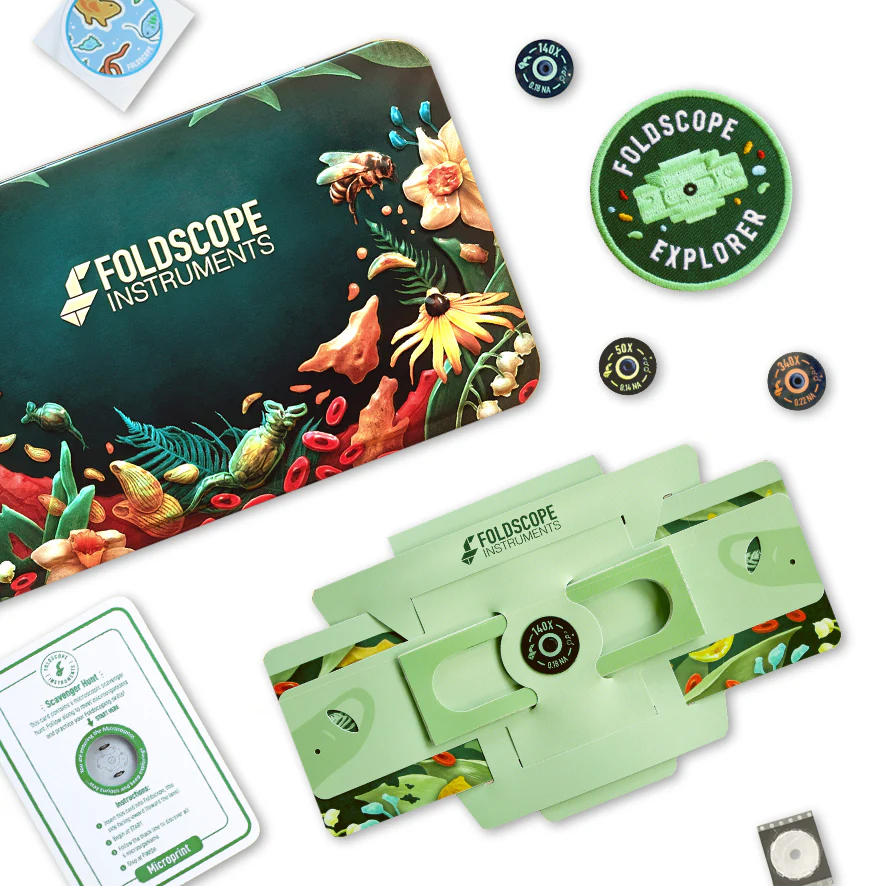

Supplement: Supplement 3 [file media-3.tif]
